# Association of glucose-lowering drugs with incident stroke and transient ischaemic attacks in primary care patients with type 2 diabetes: disease analyzer database

**DOI:** 10.1007/s00592-022-01943-7

**Published:** 2022-08-06

**Authors:** Wolfgang Rathmann, Karel Kostev

**Affiliations:** 1 Institute for Biometrics and Epidemiology, German Diabetes Center, Leibniz Center for Diabetes Research at Heinrich Heine University, Auf’m Hennekamp 65, 40225 Düsseldorf, Germany; 2 Epidemiology, IQVIA, Frankfurt, Germany

**Keywords:** Type 2 diabetes, Stroke, Transient ischaemic attack, GLP-1 receptor agonists, SGLT2-inhibitors

## Abstract

**Aims:**

Previous observational studies on glucose-lowering drugs and risk of stroke in type 2 diabetes yielded conflicting results. The aim was to examine the association of glucose-lowering drugs with incident stroke and transient ischaemic attacks (TIA) in newly diagnosed type 2 diabetes.

**Methods:**

We conducted a retrospective cohort analysis of the disease analyzer, which comprises a representative panel of 1248 general and internal medicine practices throughout Germany (01/2000–12/2019: 9.8 million patients). Incident non-fatal stroke/TIA was defined based on ICD-10 codes (I63, I64; G45) in newly diagnosed type 2 diabetes. Cox regression models were fitted to obtain hazard ratios (HR; 95%CI) for stroke/TIA adjusting for potential confounders (age, sex, health insurance, coronary heart disease, myocardial infarction, heart failure, polyneuropathy, blood pressure, eGFR) and anthropometric and metabolic intermediators (BMI, HbA1c, HDL- and LDL-cholesterol, triglycerides, lipid-lowering drugs).

**Result:**

312,368 persons with newly diagnosed type 2 diabetes without previous stroke/TIA (mean age: 64 years; 52% males) were included. There were 16,701 events of non-fatal stroke/TIA corresponding to an incidence rate of 9.3 (95%CI 9.1–9.4) per 1000 person-years. Using Cox regression, adjusted HR for stroke/TIA (per 1 year of treatment) of 0.59 (0.54–0.64) for SGLT2 inhibitors and of 0.79 (0.74–0.85) for GLP-1 receptor agonists were estimated. DPP-4 inhibitors (0.84; 0.82–0.86), metformin (0.90; 0.89–0.91), insulin (0.92; 0.91–0.93) and sulfonylureas (0.98; 0.96–0.99) also showed moderately reduced HR for stroke/TIA. Sex-specific regression analyses yielded similar results (HR).

**Conclusions:**

Treatment with SGLT2 inhibitors or GLP-1 receptor agonists might reduce non-fatal stroke/TIA in persons with newly diagnosed type 2 diabetes.

**Supplementary Information:**

The online version contains supplementary material available at 10.1007/s00592-022-01943-7.

## Background

Stroke is a major cause of premature death and disability worldwide [1]. Due to the aging population, the number of patients with stroke or transient ischaemic attacks will increase over the next decades [1]. Main modifiable risk factors for primary stroke prevention are hypertension, hyperlipidemia, smoking and type 2 diabetes [1]. Reducing the global burden of stroke requires effective preventive strategies to address such major risk factors.

Type 2 diabetes is associated with an increased risk of both ischaemic and hemorrhagic stroke [2]. A Mendelian randomization study suggested a causal association between genetic predisposition to type 2 diabetes and large artery stroke [3]. Therefore, the future worldwide increase of type 2 diabetes is a severe threat for stroke prevention [2]. In 2021, global diabetes prevalence (age group 20–79 years) was estimated to be 10.5% (537 million people), rising to 12.2% (783 million) in 2045 [4].

Glucose-lowering agents may be effective in primary stroke prevention in people with type 2 diabetes [5]. A meta-analysis of 48 randomized trials (RCT) indicated that the risk ratio of stroke was 0.85 (95%CI 0.77–0.94) for GLP-1 receptor agonists (GLP1-RA) and 0.82 (0.69–0.98) for thiazolidinediones [5]. Metformin had a borderline relative risk of 0.66 (0.42–1.06), however, based on small studies with few events. Sulfonylureas, dipeptidyl peptidase-4 inhibitors (DPP-4i) and SGLT2 inhibitors (SGLT2i) lacked evidence of reduced stroke risk in RCT [5]. However, a major limitation of this meta-analysis was the heterogeneity of stroke diagnoses used in the various trials [5]. Some studies stringently diagnosed ischaemic and haemorrhagic stroke, but others only differentiated between fatal and non-fatal stroke, which may include both forms of stroke [5].

Results from real-world studies on the association between glucose-lowering drugs and risk of stroke in type 2 diabetes have been conflicting [6, 7]. Major limitations are insufficient control of confounding variables and heterogeneity in glucose-lowering treatment duration and dosages. Nevertheless, results of observational studies were encouraging, indicating a moderate reduction in risk of stroke associated with glucose-lowering drugs, in particular, for GLP-1RA and SGLT2i [6, 7].

Therefore, the aim of our study was to examine the association of glucose-lowering drugs with non-fatal stroke/TIA in people with newly diagnosed type 2 diabetes in general, internal and diabetologist practices. Our goal was to complement results from randomized clinical trials with a broader population of persons with type 2 diabetes treated in primary care.

## Methods

### Disease analyzer database

The disease analyzer (DA) is a computerized health care database, which comprises a representative panel of physicians’ practices in Germany [8]. The DA assembles drug prescriptions, diagnoses, and basic medical and demographic data directly obtained from the practice computer system. The database has been shown to provide valid estimates of incidence and prevalence of type 2 diabetes, as well as frequencies of other chronic diseases [8]. Good agreement between the DA and German reference data was also found for prescription of glucose-lowering drugs [9].

### Study sample

**Fig. 1:**
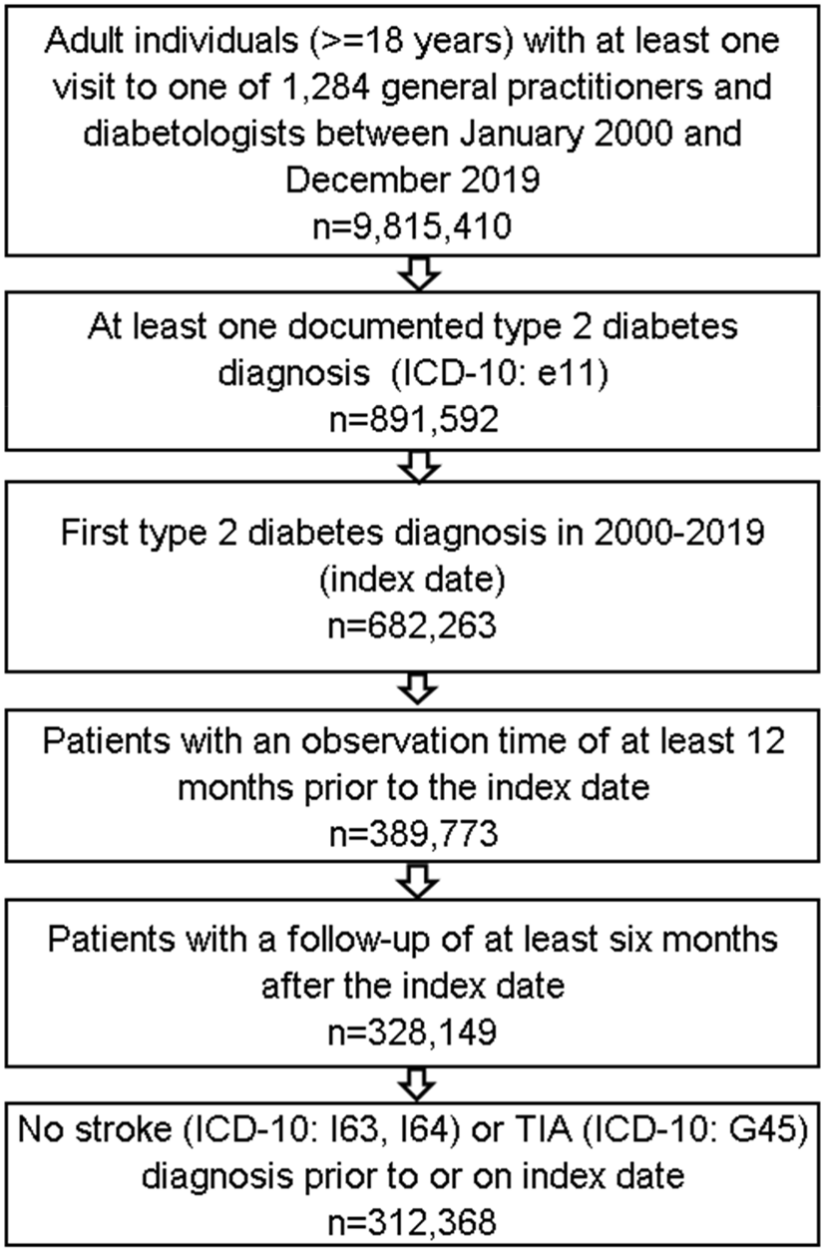
Selection of study patients: disease analyzer database

For the present study, we used completely anonymized DA data from 1284 general practitioners and internists (including diabetologists) on adult patients with ≥ 1 visit between January 2000 and December 2019 (*N* = 9.8 million patients). First, all cases with newly diagnosed type 2 diabetes (ICD 10: E11) (index date) were detected (Fig. 1). After restricting on adult persons with an observation time of ≥ 12 months before and ≥ 6 months after index dates, 328,149 people with newly diagnosed type 2 diabetes were selected. After excluding patients with previous strokes (ICD-10: I63, I64) or transient ischaemic attacks (G45), 312,368 patients were finally included in the study (Fig. 1).

### Statistical analyses

Descriptive statistics were reported as means (standard deviation [SD]), median (interquartile range) or numbers (percentage). Variables with skewed distributions were log-transformed. To analyze association of glucose-lowering drugs with incident stroke/TIA, hazard ratios (HR) with corresponding 95% confidence intervals were estimated using Cox regression (dependent variable: first event of stroke or TIA). First, age- and sex-adjusted regression models were fitted. Then, additional adjustment for potential confounders (health insurance, coronary heart disease, myocardial infarction, heart failure, polyneuropathy, systolic and diastolic blood pressure, eGFR) was carried out. Finally, potential anthropometric and metabolic intermediating variables (BMI, HbA1c, HDL-cholesterol, LDL-cholesterol, triglycerides) and therapy with lipid lowering drugs were included in the models. Models were first separately fitted for each drug class and finally a combined model was fitted including all drug groups. Finally, sex-specific models were fitted. All statistical analyses were performed with SAS (version 9.4; SAS Institute, Cary, USA).

## Results

### Clinical characteristics

Mean age of people newly diagnosed with type 2 diabetes between 2000 and 2019 in general or internal practices (*n* = 312,368) was 64 years (52% males) (Table 1). Overall, 51% were treated with metformin during the first years after diagnosis, followed by DPP-4i (22%). SGLT2i, sulfonylureas and GLP-1RA were less often prescribed. About 30% already received insulin prescriptions during the first years after diabetes diagnosis. Average HbA1c in those with recorded values was 6.8%. Cardiovascular risk factors (hypertension, hyperlipidemia) were frequently present and prior coronary heart disease and kidney disease was documented in about a quarter (Table 1). About one third had diagnosed polyneuropathy and peripheral vascular disease was present in 12%. Heart failure was diagnosed in 16% and history of myocardial infarction was found in 8%.

**Table 1:** Demographic and clinical characteristics of primary care patients with type 2 diabetes: disease analyzer database (*N* = 312,368)

Variable	*N* (%), means (SD) or median (Q1, Q3)
*Age (in years)*	
Mean (standard deviation)	64.4 (13.0)
18–50 years	45,994 (14.7)
51–60 years	68,991 (22.1)
61–70 years	85,472 (27.4)
71–80 years	78,893 (25.3)
> 80 years	33,018 (10.6)
*Sex*	
Female	150,726 (48.3)
Male	161,642 (51.8)
Private health insurance	16,860 (5.4)
*Comorbidities documented prior to the diagnosis of stroke or the end of follow-up (ICD-10 codes)*	
Hypertension (I10)	237,092 (75.9)
Lipid metabolism disorders (E78)	169,186 (54.2)
Polyneuropathy (E11.4 and G63)	91,708 (29.4)
Coronary heart disease (I24 and I25)	82,089 (26.3)
Kidney disease (E11.2, N18 and N19)	75,553 (23.2)
Peripheral vascular disease (E11.5, I70.2 and I73.9)	38.366 (12.3)
Myocardial infarction (I21, I22 and I23)	24,656 (7.9)
Heart failure (I50)	49,393 (15.8)
*Clinical characteristics documented prior to the diagnosis of stroke or the end of follow-up (mean, standard deviation)*	
Body mass index (kg/m^2^) (*n* = 121,365)	30.8 (6.3)
Systolic blood pressure (mmHg) (*n* = 107,105)	137.7 (20.6)
Diastolic blood pressure (mmHg) (*n* = 107,105)	80.3 (11.4)
HbA1c (%) (*n* = 206,673)	6.8 (1.3)
Total cholesterol (mg/dl) (*n* = 216,645)	192.1 (128.4)
HDL cholesterol (mg/dl) (*n* = 195,818)	50.6 (14.3)
LDL cholesterol (mg/dl) (*n* = 190,352)	117.2 (40.3)
Triglycerides (mg/dl) (*n* = 195,843)	81 (68–98)
eGFR (ml/min/1.73 m^2)^ (CKD-EPI equation) (*n* = 213,122)	96.5 (64.4–135.5)
*Glucose-lowering drugs prior to the diagnosis of stroke or the end of follow-up (n; %)*	
Metformin	158,914 (50.9)
Sulfonylurea	35,051 (11.2)
DPP-4 inhibitors	68,155 (21.8)
GLP-1 receptor agonists	21,282 (6.8)
SGLT2 inhibitors	35,338 (11.3)
Insulin	92,881 (29.7)
*Other drugs documented prior to the diagnosis of stroke or the end of follow-up (n; %)*	
Antihypertensives	219,181 (70.2)
Lipid-lowering drugs	129,857 (41.6)
Antithrombotic agents	57,080 (18.2)

Data are means (SD), median (Q1, Q3) or proportions

*ICD-10* International classification of diseases, *10th revision* HbA1c glycosylated hemoglobin, *HDL* high-density lipoprotein, *LDL* low-density lipoprotein, *eGFR* estimated glomerular filtration rate, *CKD-EPI* chronic kidney disease epidemiology collaboration

The average BMI, which was documented in about one third, was 31 kg/m^2^ (Table 1). Blood pressure was also documented in one third, whereas lipid values and eGFR were measured in about two thirds of the patients (Table 1). In particular, the recorded average systolic blood pressure was high. Overall, 70% of the patients received antihypertensives (ATC: C02, C03, C07, C08, C09) and 42% were treated with lipid-lowering drugs (C10). Platelet inhibitors and new oral anticoagulants (B01) were used in 18% before first stoke/TIA event.

### Incidence of stroke/TIA

We identified 16,701 events of first non-fatal stroke/TIA during 4.9 years (mean) of follow-up, corresponding to an incidence rate of 9.3 (95%CI 9.1–9.4) per 1000 person-years. Age- and sex-specific incidence rates of documented stroke/TIA in newly diagnosed type 2 diabetes are shown in Table 2. Incidence rates increased with higher age in both sexes. In all age-groups, higher incidence rates of stroke/TIA were observed in men than in women, except for the age group above 80 years, where similar rates were found in both sexes.

**Table 2:** Number of stroke/TIA events and incidence rates by sex and age-groups in 312,368 people with newly diagnosed type 2 diabetes: Disease Analyzer database

Age (years)	Events (*n*) men	Events (*n*) women	Person-years at risk men	Person-years at risk women	Incidence rates (95%CI) per 1000 PY (Men)	Incidence rates (95%CI) per 1000 PY (women)
All ages	8790	7911	922,693	876,664	9.5 (9.3–9.7)	9.0 (8.8–9.2)
18–50	507	303	154,762	120,154	3.3 (3.0–3.6)	2.5 (2.3–2.8)
51–60	1586	899	242,995	183,311	6.5 (6.2–6.9)	4.9 (4.6–5.2)
61–70	2785	1959	281,662	251,861	9.9 (9.5–10.3)	7.8 (7.4–8.1)
71–80	2973	3073	198,724	238,491	15.0 (14.4–15.5)	12.9 (12.4–13.4)
> 80	889	1677	44,551	82,847	20.0 (18.7–21.3)	20.2 (19.3–21.2)

*PY* person-years

### Association between glucose-lowering drugs and stroke/TIA

**Fig. 2:**
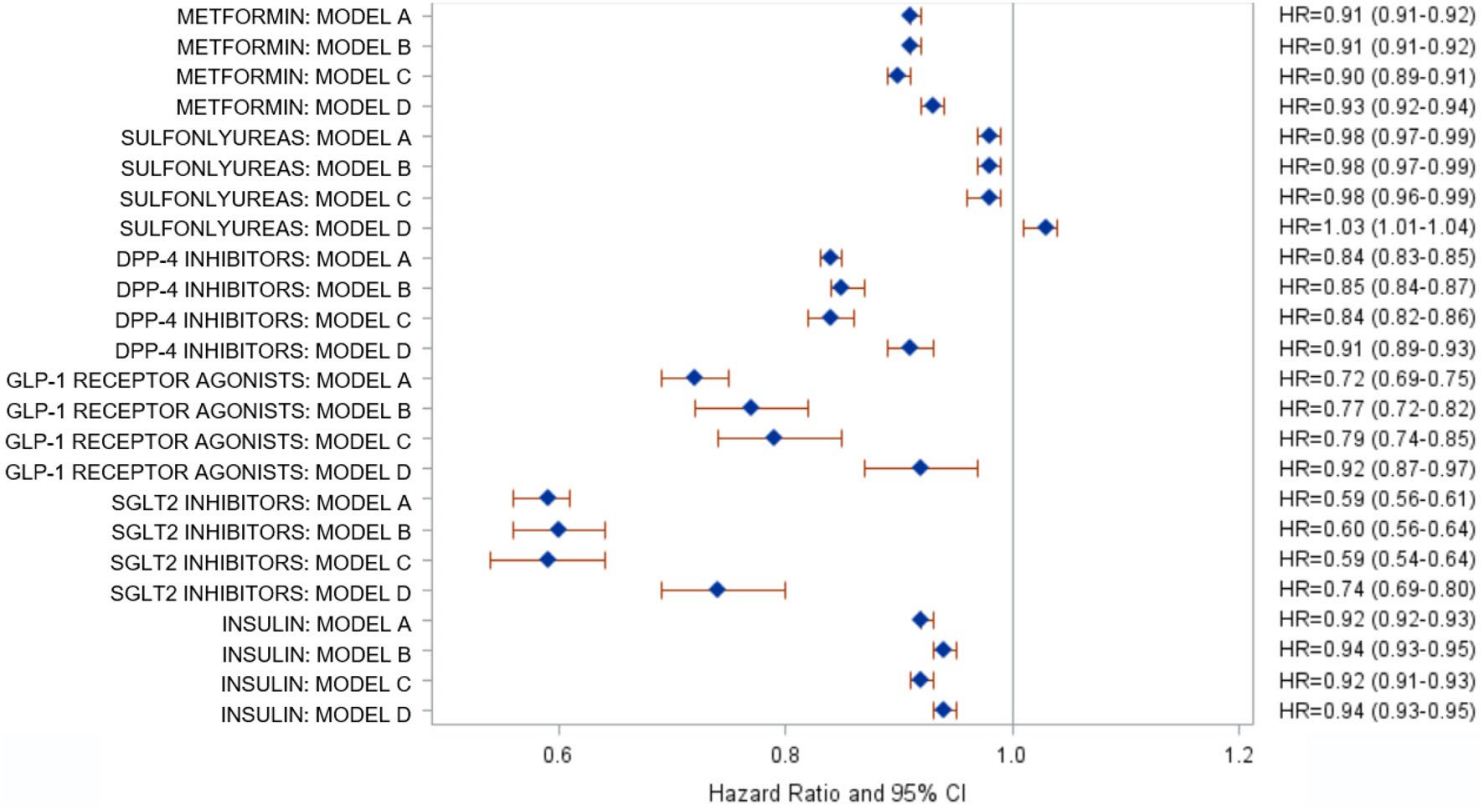
Association of glucose-lowering drugs with incident stroke/TIA in newly diagnosed type 2 diabetes patients: adjusted hazard ratios per year of drug therapy (95% CI). Cox regression models; model A: adjusted for age and sex; model B: model A and confounders (health insurance, coronary heart disease, myocardial infarction, heart failure, polyneuropathy, systolic and diastolic blood pressure, eGFR); model C: model B and anthropometric and metabolic intermediators (BMI, HbA1c, HDL-cholesterol, LDL-cholesterol, triglycerides), lipid lowering drugs Model D: all glucose-lowering drugs included together in model C. Model A (*n* = 312,368); Model B (*n* = 94,007); Model C (*n* = 63,900); Model D (*n* = 63,900). Triglycerides and eGFR were ln-transformed

Cox regression models were used to investigate the association of various glucose-lowering drugs with incident stroke/TIA (Fig. 2). Information on glucose-lowering therapy was available in all patients (*n* = 312,368). The age- and sex-adjusted HR for stroke/TIA were 0.72 for GLP-1RA and 0.59 for SGLT2i (Fig. 2). After further adjustment for confounders and potential anthropometric and metabolic intermediators in persons with complete data (*n* = 63,900), HR were 0.79 (95%CI 0.74–0.85) for GLP-1RA and 0.59 (0.54–0.64) for SGLT2i, respectively. DPP-4i were also related to a lower HR (0.84; 0.82–0.86). For other glucose-lowering drugs, also slightly to moderately reduced HR below 1.0 were found: metformin (0.90; 0.89–0.91), insulin (0.92; 0.91–0.93) and sulfonylureas (0.98; 0.96–0.99). Fully adjusted models (model D) was fitted including all glucose-lowering drugs simultaneously entered. All HR for the drug groups increased towards 1.0, but SGLT2i and GLP-1RA were still related a lower HR for stroke/TIA (Fig. 2). After adjusting for all other glucose-lowering drugs, sulfonylureas were associated with a slightly increased HR (1.03; 1.01–1.04).

Antihypertensives and antithrombotic agents were additionally included into the full model (data not shown). The reduced HR for GLP-1RA and SGLT2i persisted, as well as the HR for the other glucose-lowering agents. Finally, sex-specific models were fitted (Suppl. Figure S1). Adjusted HR for all glucose-lowering drugs were largely similar in both men and women with overlapping 95% confidence intervals.

## Discussion

In the present real-world study, we investigated the association between glucose-lowering drugs and incident stroke/TIA in persons with newly diagnosed type 2 diabetes. The key findings were that both GLP-1RA and SGLT2i were associated with lower HR for stroke indicating potential protective effects in both men and women. Observational studies complement the results of randomized controlled trials (RCT) because they include more typical persons with type 2 diabetes in a real-world health care setting.

### GLP-1RA and stroke

A network meta-analysis of 765 RCT indicated that both GLP-1RA and SGLT2i, when added to other glucose-lowering drugs, reduce mortality, non-fatal myocardial infarction, and kidney failure in people with type 2 diabetes [10]. GLP-1RA also reduced risk of non-fatal stroke [10]. Previous observational studies have also shown that GLP-1RA are related to a lower risk of stroke in patients with type 2 diabetes [11–14].

A retrospective cohort study among 132,737 insured adults with type 2 diabetes who started a second-line glucose-lowering drug after taking metformin used US nationwide administrative claims data [11]. Treatment with GLP-1RA was associated with a lower HR for stroke (0.65; 95%CI 0.44–0.97) compared to DPP-4i [11]. Combined exenatide and insulin treatment was associated with a similar lower HR for stroke (0.65; 95%CI 0.44–0.98) compared to insulin therapy alone in another database analysis [12]. Furthermore, a large database study from the US confirmed that GLP-1RA exposure was associated with a reduction in the risk of cardiovascular events including stroke both among type 2 diabetes patients with and without established cardiovascular disease [13]. Data from nationwide registers in Denmark and Sweden were used to investigate incident users of liraglutide or DPP-4i, matched 1:1 on age, sex, and propensity score [14]. Compared with DPP-4i, there was an indication for a lower HR for stroke (0.88; 0.77–1.01) in liraglutide users, although not statistically significant [14]. In a retrospective cohort study from the Taiwan National Health Insurance Research Database the risk of hospitalization for ischemic stroke for GLP-1RA users was also not statistically significantly lower than that for nonusers, although the HR indicated a protective effect (HR: 0.69; 95% CI 0.47–1.00) [15]. Thus, most previous observational studies from the US, Europe and Asia confirm our present results that GLP-1RA are associated with a lower HR for stroke after adjusting for confounders and modifiers in people with type 2 diabetes.

GLP-1RA are gaining increased attention as possible neuroprotective agents [16]. The GLP-1 receptor is widely expressed in the brain and GLP-1RA may cross the blood–brain barrier [16]. Therefore, the GLP-1 receptor is a promising therapeutic target for central nervous system diseases including stroke [16]. An experimental study showed that the GLP-1RA exendin-4 reduced neurologic deficit scores and infarct areas, and ameliorated blood–brain barrier breakdown in rats after cerebral artery occlusion [17]. The GLP-1RA liraglutide exerted neuroprotective activities, including suppressing oxidative stress, promoting cell growth, inhibiting apoptosis, and reducing inflammatory responses in cerebral ischemia–reperfusion injury in mice [18]. Thus, there is a growing experimental evidence that GLP-1RA are potential neuroprotective agents, supporting the present observational study.

### SGLT2i and stroke

The meta-analysis of RCTs indicated that SGLT2i appeared to have no effect on risk of stroke in type 2 diabetes [10]. In contrast, a systematic review of observational studies showed that SGLT2i reduced the chance of having strokes in type 2 diabetes based on fourteen cohort studies enrolling 3.2 million patients [7]. In line with the present investigation, SGLT2i lowered the odds of having stroke compared with other glucose-lowering drugs (odds ratio, OR: 0.75; 95% CI 0.72–0.78) [7]. Study participants came from various regions (Nordic countries, United States, Asia, Middle East), covering different insurance types, socioeconomic positions, drug adherence, and risk factors for cardiovascular disease [7]. In addition, there was a wide range of previous cardiovascular events from 11 to 33% in the study participants [7]. It is noteworthy, that a lower OR of stroke for SGLT2i was consistently observed irrespective of different regions or previous cardiovascular events [7].

A recent review provided a possible explanation for the opposite observed effects of SGLT2i on stroke in RCTs and observational studies [19]. This meta-analysis included five SGLT2i trials: EMPA-REG OUTCOME (Empagliflozin, Cardiovascular Outcomes, and Mortality in Type 2 Diabetes), CANVAS (Canagliflozin and Cardiovascular and Renal Events in Type 2 Diabetes), DECLARETIMI 58 (Dapagliflozin and Cardiovascular Outcomes in Type 2 Diabetes), CREDENCE (Canagliflozin and Renal Outcomes in Type 2 Diabetes and Nephropathy), and VERTIS CV (Cardiovascular Outcomes with Ertugliflozin in Type 2 Diabetes) [19]. The meta-analysis showed that for ischemic stroke, SGLT2i treatment had a neutral effect (Relative Risk: 0.99; 95%CI 0.88–1.11), without any significant heterogeneity between the trials [19]. However, when only hemorrhagic strokes were included, which were analyzed in three RCTs (EMPA-REG, CANVAS, CREDENCE), treatment with SGLT2i was associated with a 50% risk reduction (RR: 0.49; 95%CI 0.30–0.82) [19].

The strong protective effects of SGLT2i on hemorrhagic stroke may be attributed to blood pressure reduction [20]. SGLT2i decrease blood pressure by multiple mechanisms including diuresis, glucouresis, natriuresis, dehydration and weight loss [21]. Blood pressure lowering is associated with a decreased risk of stroke, especially for the hemorrhagic subtype [22]. Type 2 diabetes is mainly associated with an increased risk of ischemic stroke, but its contribution to other stroke subtypes is largely unknown [3, 23]. Most observational studies, which were based on electronic health records, combined hemorrhagic, ischemic, and other types of stroke [24]. Thus, further prospective studies are needed to compare the effects of SGLT2i on different stroke subtypes in type 2 diabetes.

Furthermore, renal function should be evaluated as potential effect modification factor in the association between SGLT2i and stroke. In the CANVAS program and the CREDENCE trial there was a significant risk reduction of stroke for participants with lower eGFR [19]. Most findings in RCTs about the effects of SGLT2 inhibitors on stroke came from subjects with normal renal function. Further research is warranted to explore the association of SGLT2i with stroke and its subtypes in people with type 2 diabetes and impaired renal function [19].

Finally, both SGLT1 and SGLT2 receptors are expressed in the human central nervous system and SGLT2i are lipid-soluble and may cross the blood–brain barrier [25]. After brain injury in a murine model, SGLT1 blockage showed beneficial effects with regard to area and volume of the lesions, edema, and motoric disability [25]. Sotagliflozin is a novel dual SGLT1/SGLT2 inhibitor, which reduced total fatal or non-fatal stroke (HR: 0.66; 95%CI 0.48–0.91) in a post hoc analysis in persons with type 2 diabetes and chronic kidney disease [26]. A network meta-analysis of 14 RCT, including the recent SCORED and SOLOIST trials investigating the effects of sotagliflozin, showed that among gliflozins, sotagliflozin had the greatest effect in lowering risk of stroke [27]. Thus, larger studies are needed to confirm the effects of SGLT1/SGLT2 inhibition on stroke in type 2 diabetes and to elucidate the potential mechanisms [28].

### Sulfonylureas, DPP4i, insulin, and stroke

In the present study, a slightly increased HR for stroke related to sulfonylurea therapy was found in the full model adjusting for all glucose-lowering drugs. However, this small increase (HR: 1.03) appears not to be clinically relevant. Observational studies have reported conflicting results regarding the association of sulfonylurea treatment with risk of cardiovascular events including stroke [5, 29, 30]. There is still an intense debate surrounding safety issues of sulfonylureas, especially in the context of current cardiovascular outcome trials [31]. Based on 23 RCTs, a meta-analysis found no significant association between sulfonylureas and stroke (OR 1.16; 95%CI 0.81–1.66) [29]. However, as in the present analysis, the effect estimate was slightly increased [29]. It is noteworthy, that the meta-analysis as well as the present study, was not specifically designed to compare second- and third-generation sulfonylureas, which have different risks of hypoglycemia [29]. Thus, further studies are needed to guide decision making about the place of sulfonylureas in contemporary glucose-lowering therapy [31]. The use of sulfonylureas is decreasing in high-income countries, even though they are still prescribed in low- and middle-income regions.

In the present observational study, insulin therapy showed an almost neutral effect on stroke risk in the final model. Insulin is often used in people with type 2 diabetes when glycemic control is still insufficient despite up-titration to maximal doses of dual therapies, which are usually metformin plus sulfonylureas or DPP-4i [32]. Limited data are available on stroke risk and other cardiovascular outcomes in various third-line glucose-lowering therapies, e.g. insulin and GLP-1RA [32]. Insulin is associated with weight gain, which may indirectly increase stroke risk, whereas adding GLP-1RA has favorable effects on weight reduction [32]. A database study from the UK showed that intensification of glucose-lowering therapy with GLP-1RA was associated with a 73% reduction in the risk of composite cardiovascular events, including stroke, compared to insulin [32]. It is noteworthy, that the HbA1c reduction was similar in both groups, but a large difference in weight response was observed, i.e. weight gain with insulin and weight reduction with GLP-1RA [32]. Risk of stroke was 61% lower in the GLP-1RA cohort, although this was not statistically significant [32]. Thus, further real-world studies are required to study stroke risk in type 2 diabetes following intensification of glucose-lowering dual or triple therapies with various drugs [32, 33].

Finally, the role of DPP-4i as an add-on therapy to metformin needs to be further determined [34]. In the present study a moderately lower HR for stroke was found for DPP-4i, which was reduced after controlling for other glucose-lowering drugs. Cardiovascular safety trials suggest that DPP-4i do not increase risk of major cardiovascular events, including stroke compared to placebo or to sulfonylureas [34]. A database study in the UK included 17,570 type 2 diabetes patients with sulfonylureas and 6267 with DPP-4i as add-on to metformin with a similar prevalence of comorbidities [34]. The HR for stroke was 1.0 (95%CI 0.81–1.25) comparing sulfonylureas and DPP-4i cohorts [34]. Thus, cardiovascular considerations (including stroke) may not be the basis for choosing between DPP-4i or sulfonylureas as second-line glucose-lowering therapy.

### Study strengths and limitations

Our study has several strengths. First, the DA database has been shown to be largely representative for diagnoses and drug prescriptions of several chronic diseases, including type 2 diabetes [8, 9]. As an example, the estimated incidence rate of non-fatal stroke/TIA in type 2 diabetes and the relationship with age and sex were comparable to a representative practice-based study from UK (DA: 9.3 per 1000 person-years; 95%CI 9.1–9.4; General Practice Research Database: 11.9 per 1000 person-years; 95%CI: 11.4–12.4) [35]. The slightly higher incidence rate in the UK database is most likely due to inclusion of early deaths within 30 days after stroke [35]. Second, the sample size of the present study was larger than many previous investigations on the same topic. Third, anonymized patient data was collected by medical doctors in primary care so that recall bias was unlikely. A main limitation is that there are many missing data for laboratory values and clinical measures (e.g. blood pressure, BMI). The DA only includes data assessed by general and internal medicine physicians in their daily clinical practice. However, this also gives important information on patterns of routine practice. Furthermore, information on individual dosage of glucose-lowering drugs was not complete and was therefore not analyzed. Assessment of comorbidity mainly relied on ICD-10 codes by primary care physicians only. Documentation of retinopathy in the Disease Analyser primary care database is low. Only in about 2% of the patients, diagnoses by ophthalmologists are documented. However, both neuropathy and eGFR were analyzed to reflect severity of microvascular complications. Finally, data on socioeconomic status (e.g. education, income) and lifestyle-related risk factors (e.g. smoking, alcohol, physical activity) were lacking.

## Conclusions

In conclusion, treatment with GLP-1RA and SGLT2i may reduce non-fatal stroke or TIA in newly diagnosed type 2 diabetes patients. If confirmed, these real-world findings should be considered by physicians when selecting among the various alternative glucose-lowering drugs for their patients with type 2 diabetes.

## Supplementary Information

Below is the link to the electronic supplementary material.

Supplementary file1 (DOCX 344 kb)

## Data Availability

The disease analyzer data are not publicly available due to confidentiality issues. Investigators should contact IQVIA (Frankfurt, Germany) to ask about data availability.
