# Correction to: Association of glucose‑lowering drugs with incident stroke and transient ischaemic attacks in primary care patients with type 2 diabetes: disease analyzer database

**DOI:** 10.1007/s00592-022-01962-4

**Published:** 2022-09-06

**Authors:** Wolfgang Rathmann, Karel Kostev

**Affiliations:** 1 Institute for Biometrics and Epidemiology, German Diabetes Center, Leibniz Center for Diabetes Research at Heinrich Heine University, Auf’m Hennekamp 65, 40225 Dusseldorf, Germany; 2 IQVIA, Frankfurt, Germany

## Correction to: Acta Diabetologica 10.1007/s00592-022-01943-7

Authors would like to correct an error in Abstract.

I53 must be I63

The original article has been corrected.

