# Supplementary material for: Association of glucose-lowering drugs with incident stroke and transient ischaemic attacks in primary care patients with type 2 diabetes: disease analyzer database
Source: Acta Diabetol. 2022 Aug 6;59(11):1443–51. doi: 10.1007/s00592-022-01943-7 (PMC9519725; doi:10.1007/s00592-022-01943-7)
Supplement: Supplementary file 1 — Supplementary file1 (DOCX 344 kb) [file 592_2022_1943_MOESM1_ESM.docx]

**Figure suppl 1** Sex-specific Associations of glucose-lowering drugs with incident stroke/TIA in newly diagnosed type 2 diabetes patients: adjusted hazard ratios per year of drug therapy (95% CI)
**Women**


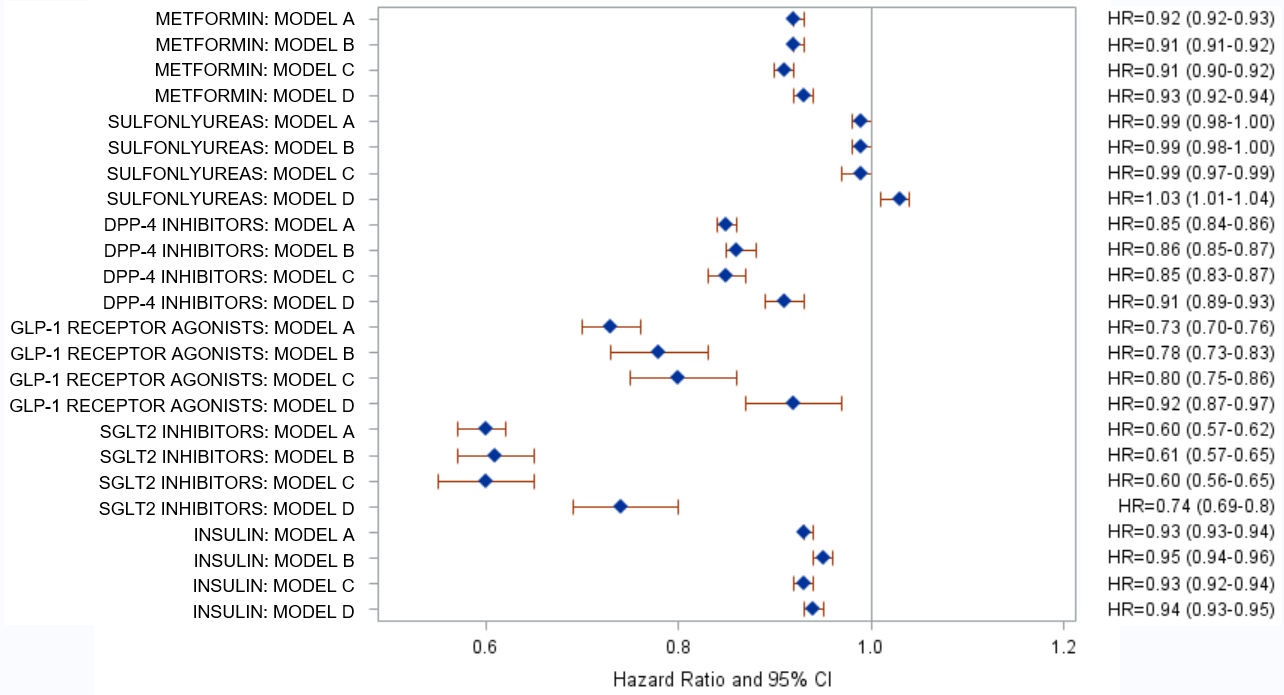


**Men**


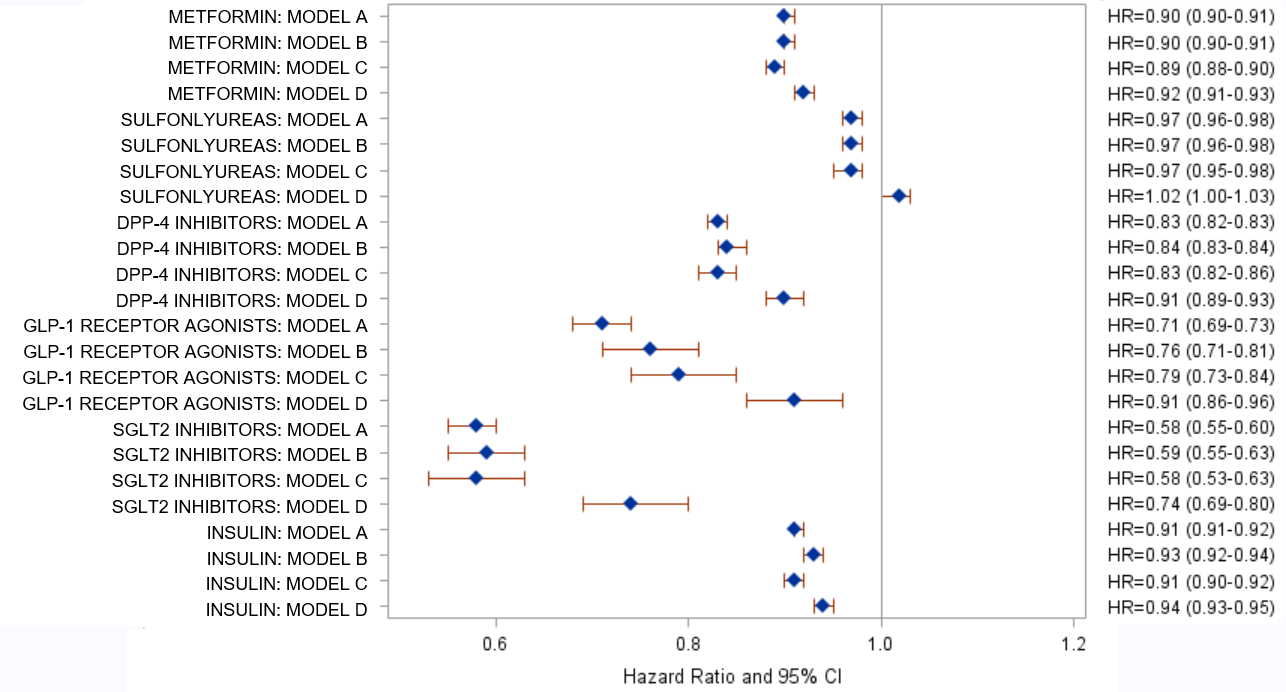


Cox regression models; model A: adjusted for age; model B: model A and confounders (health insurance, coronary heart disease, myocardial infarction, heart failure, polyneuropathy, systolic and diastolic blood pressure, eGFR) model C: model B and anthropometric and metabolic intermediators (BMI, HbA1c, HDL-cholesterol, LDL-cholesterol, triglycerides), lipid lowering drugs
Model D: all glucose-lowering drugs included together in model C
